# Will the latest wave of the COVID‐19 pandemic be an ecological disaster? There is an urgent need to replace plastic by ecologically virtuous materials

**DOI:** 10.1002/hsr2.703

**Published:** 2022-08-07

**Authors:** Jean‐Michel Mansuy, Marion Migueres, Pauline Trémeaux, Jacques Izopet

**Affiliations:** ^1^ Laboratoire de virologie CHU Toulouse, Hôpital Purpan, Institut fédératif de Biologie Toulouse France; ^2^ Institut Toulousain des Maladies Infectieuses et Inflammatoires (Infinity) INSERM UMR1291—CNRS UMR5051 Toulouse France; ^3^ Université Toulouse III Paul‐Sabatier Toulouse France

**Keywords:** antigen, COVID‐19, diagnosis, ecology, PCR, plastic

## Abstract

**Background and Aims:**

Direct virological diagnosis of Severe Acute Respiratory Syndrome CoronaVirus 2 (SARS‐CoV‐2) infectionis based on either viral antigen or viral genome detection. These methods, in addition to the dedicated reagents and transport packaging, require the use of quantities of plastic that may individually appear negligible but which, in the context of a pandemic, are very high. The aim was to estimate the amount of plastic involved in a diagnostic assay whether molecular or antigenic.

**Methods:**

We weighed the plastics used to obtain a diagnostic assay result for SARS‐CoV‐2 infection in our hospital.

**Results:**

Each ready‐to‐use antigen assay requires about 20 g of plastic whereas the PCR assay implies the use of 30 g. This unit mass, when compared to our laboratory's SARS‐CoV‐2 genomic screening activity,represents more than 10 tons of plastic for 2021. At our region level (#6.10 inhabitants), more than 350 tons of plastic were used to carry out more than 7 million declared PCR assays and as many antigenic assays.

**Conclusions:**

The virologic diagnostic activityl inked to the SARS‐CoV‐2 pandemic has highlighted once more our dependance for plastic use. We must already think about a more environmentally virtuous diagnostic activity by integrating a reasonned use of diagnostic tools and a higher use of ecological friendly material. Parallel the notion of waste management must also be addressed in order to limit their environmental impact.

The use of plastics is ecologically nonvirtuous on two distinct levels: their manufacture, which is based on fossil hydrocarbon materials and contributes to global warming by generating greenhouse gases, and their disposal, which generates major macroscopic and microscopic pollution,^1^ given the lifespan of these materials, varying from 100 to 1000 years. Thus, all the plastics produced since the beginning of their intensive use in the 1950s are still present in our environment and only 9% have entered a recycling cycle.^2^ The recent discovery of plastic microparticles and their quantification in human blood^3^ reflects this major pollution and is a new warning signal for world leaders. Faced with this environmental and health issues, many countries, whatever their income status, have established policies to reduce or eliminate the use of plastics.^4^

However, the pandemic triggered by the emergence of the severe acute respiratory syndrome coronavirus 2 (SARS‐CoV‐2) in December 2019 in Wuhan China has not only put a dramatic brake on these policies, but also generated new sources of plastic pollution, such as the increasing use of protective face masks.^4^

Soon after the beginning of the pandemic, International and National health authorities have defined and continuously updated specific criteria for testing for SARS‐CoV‐2. Testing concern, in the one hand, patients suffering from respiratory infection especially because specific symptoms of a Coronavirus Infectious Disease 2019 (COVID‐19) are rare^5^ and, on the other hand, some asymptomatic individuals for example, after close contact with an individual with COVID‐19, before surgical procedures or before receiving immunosuppressive therapy.

Nucleic acid amplification testing (NAAT)—such as polymerase chain reaction (PCR)—to detect SARS‐CoV‐2 RNA from the respiratory tract or from saliva^6^ is the diagnosis test of choice for COVID‐19.^7^ However, NAAT are expensive, require trained personnel, and dedicated premises and equipment. That is why antigen testing may be the test initial used,^8^ even if the sensitivity of such tests is lower than NAATs one.

This pandemic have resulted in close to 530,000,000 cases of COVID‐19 and over 6,200,000 deaths by May 30, 2022.^9^ It has evolved with the appearance of waves of new virus variants. The latest one's, Omicron, has rapidly spread worldwide, and was responsible of 300,000–500,000 new cases each day in France during the past 3 weeks. Counting these cases involves a molecular or antigenic diagnostic test performed by operatives wearing disposable plastic protective equipment. The test kits have plastic packaging and are transported in single‐use plastic bags.

We have used a simple, rather crude method to estimate the amount of plastic involved in a diagnostic test. It does not take the plastic used for transporting samples into account, nor that used for personal protective equipment. We just weighed the reagents used to obtain an antigen test or PCR test result in our hospital. Each ready‐to‐use antigen assay includes about 20 g of plastic. The PCR assay (plastic microwell plate: 0.2 g plastic/sample) is performed on a nasopharyngeal sample (plastic: 18 g/sample) from which nucleic acids are extracted (microwell plate: 4 g plastic/sample, micropipette tips: 7.8 g plastic/sample). The PCR process consumes 30 g plastic/sample. We performed 362,000 PCR detections in our laboratory last year (2021), which required 10,860 kg of plastic. A total of 7,002,012 PCR tests and 7,198,479 antigen tests (not counting self‐administered tests) were performed in 2021 in the Occitanie region (72,724 km², 5,933,000 inhabitants), where Toulouse University Hospital is located. They required 210 tons (PCR) and 144 tons (antigen) of plastic.

It is quite impossible to determine how many self‐administered antigen tests were carried out after contact with a COVID‐19 patient, as recommended by the French Health authorities (tests performed on days 2 and 3 postcontact if negative result on day 0). Self‐detection tests are now widely used and readily available; they are used by individuals before public or family events, following symptoms compatible with COVID‐19. Self‐administered tests are undoubtedly more numerous now than antigen tests performed by healthcare staff, accounting for a considerable weight of plastic.

Worldwide, thousands of tons of plastic have been, are being, and will be used to diagnose COVID‐19, generating a mountain of waste requiring special healthcare treatment. This is why the World Health Organization published a note in February 2022 on the risk to human and environmental health of exposure to healthcare waste and waste management systems.^10^ This threat highlights the need to improve waste management practices, both in high income and low/middle income countries.

Our study has not included the amounts of nucleic acid extraction and PCR reagents because they are, by comparison, negligible, but some of them are delivered frozen, which involves dry‐ice and single‐use insulated packaging.

We believe that it is time to reflect on our experience during this pandemic and redefine healthcare paradigms, particularly those governing the use of raw materials and the disposal of manufactured products, to ensure that they are as environmentally friendly as possible. The standardized disposal of organic products does not prevent the use of “green” raw materials that have a smaller impact on the planet. Packaging can also be recycled. Clearly, many aspects of our healthcare system should be re‐examined. We could start with a simple one, limiting materials use so as to consume no more than necessary.

## AUTHOR CONTRIBUTIONS

**Jean‐Michel Mansuy**: Conceptualization; Methodology; Resources; Writing – original draft; Writing – review and editing. **Jacques Izopet**:  Supervision; Writing – review and editing.  **Marion Migueres**: Writing – original draft; Writing – review and editing. **P. Trémeaux**: Writing – original draft; Writing – review and editing.

## CONFLICT OF INTEREST

The authors declare no conflict of interest.

### TRANSPARENCY STATEMENT

The lead author (manuscript guarantor) affirms that this manuscript is an honest, accurate, and transparent account of the study being reported; that no important aspects of the study have been omitted; and that any discrepancies from the study as planned (and, if relevant, registered) have been explained.

## Data Availability

All relevant data are included in the article.
